# Mode II Fracture Analysis of GNP/Epoxy Nanocomposite Film on a Substrate

**DOI:** 10.3390/polym13162823

**Published:** 2021-08-22

**Authors:** Shiuh-Chuan Her, Kai-Chun Zhang

**Affiliations:** Department Mechanical Engineering, Yuan Ze University, Chung-Li 320, Taiwan; s1025045@mail.yzu.edu.tw

**Keywords:** graphene nanoplatelet, mode II fracture toughness, end-notched flexure, nanoindentation test

## Abstract

Epoxy resin with excellent mechanical properties, chemical stability, and corrosion resistance has been widely used in automotive and aerospace industries. A thin film of epoxy deposited on a substrate has great application in adhesive bonding and protective coating. However, the intrinsic brittleness of epoxy with a relatively low fracture toughness limits its applications. In this work, graphene nanoplatelets (GNP) were added to the epoxy resin to enhance its toughness, hardness, and elastic modulus. A series of nanocomposites with different loadings of GNP were fabricated. Ultrasonic sonication in combination with surfactant Triton X-100 were employed to disperse GNP in the epoxy matrix. A nanocomposite film with a thickness of 0.3 mm was deposited on an Al substrate using a spinning coating technology. The hardness and elastic modulus of the nanocomposite film on the Al substrate were experimentally measured by a nanoindentation test. Analytical expression of the mode II interfacial fracture toughness for the nanocomposite film on an Al substrate with an interfacial edge crack was derived utilizing the linear elastic fracture mechanics and Euler’s beam theory. End-notched flexure (ENF) tests were conducted to evaluate the mode II fracture toughness. It was found that the hardness, elastic modulus, and mode II fracture toughness of the nanocomposite film reinforced with 1 wt % of GNP were improved by 71.8%, 63.2%, and 44.4%, respectively, compared with the pure epoxy. The presence of much stiff GNP in the soft epoxy matrix prompts toughening mechanisms such as crack deflection and crack pinning, resulting in the improvements of the fracture toughness, hardness, and elastic modulus. Microscopic observation for the nanocomposite was examined by scanning electron microscopy (SEM) to investigate the dispersion of GNPs in the epoxy matrix. The performance of a nanocomposite film deposited on a substrate was rarely studied, in particular, for the interfacial fracture toughness of the film/substrate composite structure. Utilizing the theoretical model in conjunction with the ENF experimental test presented in this study, an accurate determination of the mode II interfacial fracture toughness of film/substrate composite structure is made possible.

## 1. Introduction

Graphene was first discovered by Novoselov et al. [1] in 2004. Since then, graphene has attracted attention due to its large surface to volume ratio and unique structure (a 2D single layer of carbon atoms bonded by $sp^{2}\;$to form a hexagonal honeycomb structure). It has become one of the most promising nanofillers owing to exceptional material properties such as an ultimate strength of 130 GPa [2], an elastic modulus of 1 TPa [3], thermal [4] and electrical [5] conductivities. In recent years, carbon-based nanomaterials including carbon nanotube (CNT) and GNP have been widely used to incorporate into polymer matrix to fabricate multifunctional composites [6,7]. In comparison with other nanofillers, GNP provides better performances for the enhancements of mechanical and other functional properties [8,9,10]. Cilento et al. [11] investigated the effect of GNP content on the reinforcing efficiency of GNP/epoxy nanocomposite. Zhang et al. [12] studied the correlation between the mechanical and dielectric properties of functionalized graphene/polyurethane nanocomposites. Wang et al. [13] incorporated 0.5 vol % of GNP into poly (vinyl alcohol) (PVA), resulting in an increase of 91.1% in tensile strength and an increase of 66.7% in Young’s modulus in comparison with neat PVA. Song et al. [14] added reduced graphene oxide into epoxy resin to improve the electromagnetic interference shielding effectiveness and electrical conductivity of the epoxy composites. Shadmand et al. [15] fabricated a graphene-based microcantilever as a flow sensor to measure the velocity of water flow with a piezoresistive sensitivity of 1.22 $\Omega /\left(m\cdot s^{-1}\right)$ in the range of 0 to 0.7 m/s.

Epoxy has been considered as one of the most promising polymers, and it is widely used in a variety of applications such as adhesive bonding, protective coating, and electronic device encapsulation due to its better mechanical properties and chemical resistance [16]. However, a high degree of cross-link between the epoxy and curing agent leads to the brittle characteristic of the epoxy, limiting its applications in high-end products [17]. Therefore, many studies have been reported on toughening of the brittle epoxy. Conventional methodology for the improvement of the fracture toughness is to incorporate high stiffness fillers into epoxy such as silica [18], clay [19], CNT [20], and GNP [21]. Among them, GNP has received broad attention for the reinforcement of the epoxy matrix because of its extraordinary mechanical, thermal, and electrical properties in combination with a high specific surface area. However, it was found that the toughening effects of GNP reported in the literature were quite different. The maximum increase of the mode I stress intensity factor for epoxy/graphene oxide composites can be varied from −14% to 63% [22,23,24,25,26]. It is well known that the performance of GNP-reinforced nanocomposite heavily depends on the dispersion and interfacial interaction of GNP with epoxy matrix. It is a critical issue to uniformly disperse GNPs in an epoxy matrix, since GNPs exhibit a strong tendency to agglomerate because of the van der Waals forces and π−π bondings among the carbon atoms. Recently, various methods have been proposed to graft a functional group on the surface of GNP to enhance the dispersion and interfacial interaction between the GNP and polymer matrix. There are two main approaches for the surface modification of GNPs, including covalent functionalization and non-covalent functionalization [27,28]. The disadvantage for the covalent functionalization of GNPs is the defects induced by the functionalization, leading to a decrease of the strength of the nanocomposite [29]. The drawback for the non-covalent functionalization is that the mechanical and thermal properties of the nanocomposite may be deteriorated due to a large amount of surfactant [30]. Paramsothy [31] studied the dispersion, adhesion, and alignment of CNTs in polystyrene matrix. They found that good dispersion was achieved by low CNT content (5 wt %). Moreover, interfacial adhesion was enhanced due to chemical bonds C=C of the CNT surface and phenyl groups of adjacent polystyrene matrix.

Wang et al. [32] investigated the effects of GNP size and content on the mode I fracture toughness of GNP reinforced epoxy. They found that GNP with a small size of 0.7 um and 0.1 wt % exhibited the best improvement of 75% for the mode I fracture toughness due to the fine dispersion of small GNP in the epoxy resin. In contrast, a large size and high loading of GNP may reduce the toughening effect owing to the agglomeration. Xu et al. [33] reported an increase of 200% in mode I fracture energy by incorporating 1.0 wt % GNP into epoxy matrix with 20 wt % sulfonated polystyrene-block-poly(ethylene-co-butylene)-block-polystyrene (SSEBS) due to a good dispersion and strong interaction of GNP in the epoxy matrix. Du et al. [34] investigated the toughening mechanisms of GNP in the epoxy matrix. They found that the debonding/delamination and pullout of GNP trigger and promote local plastic deformation of matrix to dissipate more fracture energy. Qiu and Wang [35] conducted a three-point bending test to determine the mode I critical stress intensity factor of GNP-reinforced nanocomposite. Experimental results showed that the fracture toughness was increased by 41% with an addition of 0.54 vol % GNP while compared with neat epoxy. Chandrasekaran et al. [36] studied the effect of addition of three different types of nanofillers (reduced graphene oxide RGO, GNP, and MWCNT) on mode I fracture toughness of epoxy-based nanocomposites. They found that RGO provided the most significant improvement of 40% for 0.5 wt % of incorporation. Crack pinning and crack surface separation initiated from RGO contributed to the enhancement of the fracture toughness.

Most of the existing literature focused on the mode I fracture toughness. The evaluation of fracture toughness under mode II fracture is relatively more challenging while compared with mode I fracture. Mode II fracture causes the crack surfaces to slide relative to each other due to the shear force exerted on the crack tip. Ahmadi-Moghadam and Taheri [37] compared the enhancement of mode I and mode II fracture toughness on GNP-reinforced epoxy. Significant improvement was achieved in mode I fracture toughness, while there was a slight increase for mode II fracture toughness due to the relatively smaller plastic zone, larger density of microcracks, and lack of filler bridging of the nature of mode II fracture. Srivastava et al. [38] investigated the effect of GNP on mode II interlaminar fracture toughness of carbon fiber-reinforced polymer (CFRP) composites using a three-point bending ENF test. An increase of 42.5% in mode II fracture toughness was achieved with an addition of 3 wt % GNP into CFRP. Jia et al. [39] experimentally studied the mode II fracture toughness of an epoxy adhesive reinforced with GNPs using the compliance-based beam method (CBBM). It was found that the mode II fracture toughness of nanocomposites reinforced by 0.5 wt % GNP exhibited a 41% enhancement compared with neat epoxy adhesive. Azevedo et al. [40] employed cohesive zone modelling (CZM) coupled with finite element analyses to predict the mode II fracture toughness of an adhesive joint. Three different adhesives were used to investigate the effect of adhesive ductility on the strength of the adhesive joint. They found that ductile adhesives have a greater capability to withstand cleavage and peel forces.

A thin film of epoxy deposited on a substrate has a great application in adhesive bonding and protective coating. The mechanical properties of the epoxy film including the hardness, Young’s modulus, and interfacial fracture toughness are important and required intensive study to meet the safety requirement. Most of the existing literature investigated the mechanical properties of the epoxy in a bulk state using a tensile test. The performances of epoxy thin film deposited on a substrate are rarely studied, in particular, for the interfacial fracture toughness of the film/substrate composite structure. The present work investigated the mechanical properties of the epoxy in a thin film state using the nanoindentation technique. In addition, a theoretical model was proposed to evaluate the mode II fracture toughness of a nanocomposite film/substrate composite structure.

In this work, nanocomposites reinforced with various loadings of GNPs were prepared through a sonication process. Horn sonication in combination with a surfactant-assisted process leads to a good dispersion of GNPs in the epoxy matrix. A non-ionic surfactant of Triton X-100 was used to enhance the wettability and compatibility in the epoxy resin. The capability of Triton X-100 for the dispersion of CNTs in epoxy resin has been reported by Geng et al. [41]. A nanocomposite film with a thickness of 0.3 mm was deposited on an Al substrate using a spinning coating technology. The hardness and elastic modulus of the nanocomposite film were determined by nanoindentation tests. End-notched flexure (ENF) specimens were prepared under a three-point bending loading to evaluate the mode II interfacial fracture toughness of nanocomposite film/substrate composite structure. The influence of GNP concentration on the hardness, elastic modulus, and fracture toughness of the nanocomposite was investigated through a series of parametric study.

## 2. Materials and Methods

### 2.1. Preparation of GNP/Epoxy Nanocomposite

**Figure 1:**
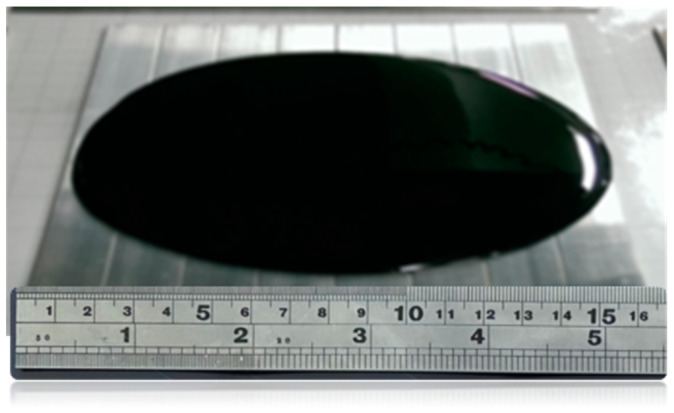
GNP/epoxy nanocomposite poured onto an Al substrate.

GNPs were obtained from Uchess Co., (New Taipei City, Taiwan) and used as received without further purification. The thickness and lateral dimension were in the ranges of 1–10 nm and 0.5–20 μm, respectively, which were provided by the manufacturer. The epoxy consists of two components, part A Mungo 4200 A and part B Mungo 4200 B, respectively, purchased from Golden Root Co., Ltd. (Taipei City, Taiwan). The epoxy was mixed with hardener at a weight ratio of 2:1 in accordance with the manufacturer’s recommendation. Ethanol was added into the liquid epoxy to reduce the viscosity, which is helpful for the dispersion. Then, GNPs were incorporated into the epoxy matrix and dispersed by a sonicator (Q700, Qsonica L.L.C., Newtown, CT, USA) The sonication probe was immerged into the GNP and epoxy mixture and operated at a pulse mode with 10 s on and 20 s off for 20 min. Thereafter, the curing agent was added into the mixture and manually stirred for 20 min. Consequently, the mixture was put in a vacuum chamber under a constant temperature of 25 °C for 60 min to remove the trapped air due to the stirring. Then, the degassed GNP/epoxy nanocomposite was poured onto an Al substrate and placed in a spinning coating machine (RMT-SC 150SS, Reliable-Mate Technology Co., Ltd., Shin-Chu City, Taiwan) as shown in Figure 1. A nanocomposite film with a thickness of 0.3 mm was deposited on an Al substrate using a spinning coating technology. The film thickness coated on the substrate can be moderated by adjusting the rotating speed of the spinning coating machine. In this work, the nanocomposite film thickness was 0.3 mm for all the test specimens. A series of nanocomposites with GNP contents of 0.3, 0.5, 0.8 and 1.0 wt % were prepared to evaluate the influence of the GNP loading on the hardness, elastic modulus, and mode II fracture toughness of the nanocomposite. The neat epoxy was also included for the comparison.

### 2.2. SEM Analysis

Microscopic observation for the nanocomposite was examined using scanning electron microscopy (SEM) to investigate the dispersion of GNPs in the epoxy matrix. For this objective, a SEM (JSM 7600F, Jeol Co., Tokyo, Japan) was used to observe the surface morphology of the nanocomposite. Due to the non-conductive characteristics of the epoxy, the specimen was first coated by platinum and worked at an accelerated voltage of 10 kV. The degree of GNP dispersion in the epoxy resin can be evaluated through the examination of the surface morphology using scanning electron microscopy.

### 2.3. Nanoindentation Tests

In this work, the mechanical properties of the hardness and elastic modulus of the nanocomposite film were determined utilizing the nanoindentation technique proposed by Oliver and Phar [42,43]. Nanoindentation tests were carried out using a nanoindenter (Nano Test, Micro Materials Ltd., Wrexham, UK), equipped with a Berkovich indenter. A typical load-indentation depth curve can be divided into three parts: loading to a maximum load, holding at the maximum load for a short period of time, and unloading back to the zero load. A holding period of 5 s was employed to eliminate the time-dependent effects.

### 2.4. Mode II Fracture Analysis

**Figure 2:**
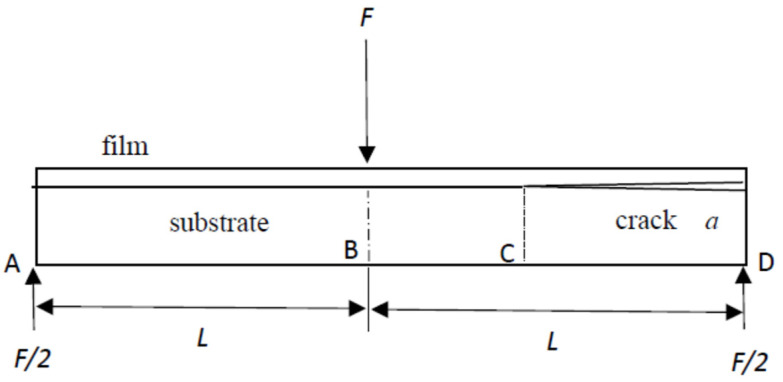
ENF specimen subjected to a three-point bending loading.

To derive the mode II interfacial fracture toughness of a film/substrate composite structure, a theoretical model based on an end-notched flexure (ENF) specimen with an edge crack along the interface was adopted. The ENF specimen was subjected to a three-point bending loading as shown in Figure 2, where F is the external load applied at the middle point B of the specimen and F/2 is the reaction force at the two supports A and D. The lengths of the edge crack and ENF specimen were *a* and 2*L*, respectively.

In this study, the strain energy release rate known as the energy available for an increment of crack extension was used to characterize the fracture toughness and derive as follows.

**Figure 3:**
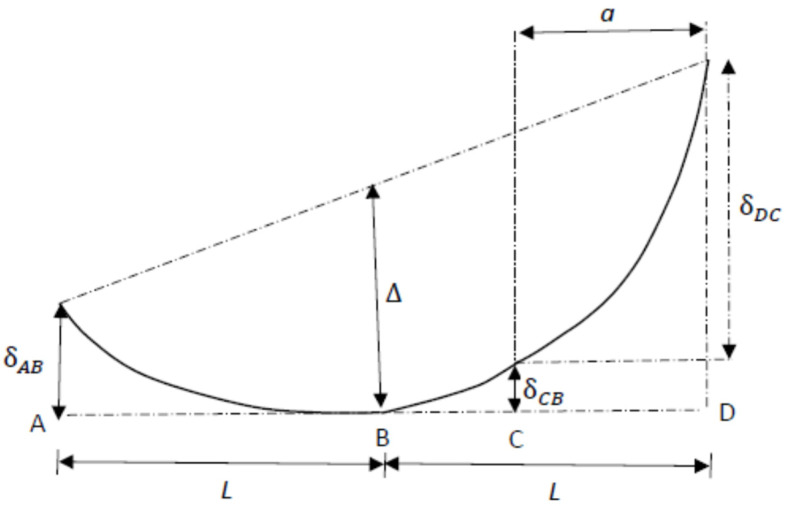
Schematic illustration of the deformation of the ENF specimen under three-point bending loading.

The deformation of the ENF specimen induced by the three-point bending loading is schematically shown in Figure 3. The displacement of point B where the external load is exerted can be written as

(1) $$\Delta =\frac{\delta_{AB}+\delta_{CB}+\delta_{DC}}{2}$$

where $\delta_{AB},\;\delta_{CB},\;\mathrm{and}\;\delta_{DC}$ are the displacements of *A*, *C*, and *D*, respectively; *C* is the tip of the edge crack, as shown in Figure 2.

*BA* and *BD* illustrated in Figure 3 were modeled as cantilever beams with a fixed end at *B* and free ends at A and D. The reaction force F/2 of the supports was exerted on the free ends A and D of the cantilever beams *BA* and *BD*. The displacements of *A* and *D* can be calculated using Euler’s beam theory as follows.

(2) $$\delta_{CB}=\frac{\frac{F}{2}{\left(L-a\right)}^{2}\left(3L-L+a\right)}{6\bar{E}\bar{I}}=\frac{F\left(2L^{3}-3aL^{2}+a^{3}\right)}{12\bar{E}\bar{I}}$$

(3) $$\delta_{AB}=\frac{\frac{F}{2}L^{3}}{3\bar{E}\bar{I}}=\frac{FL^{3}}{6\bar{E}\bar{I}}\;\;$$

(4) $$\bar{E}\;\bar{I}=\frac{b}{12}\left[E_{f}h_{f}^{3}+E_{s}h_{s}^{3}+3E_{f}E_{s}h_{f}h_{s}\frac{{\left(h_{f}+h_{s}\right)}^{2}}{E_{f}h_{f}+E_{s}h_{s}}\right]$$

where $\bar{E}\bar{I}$ is the flexural rigidity of the composite beam containing a nanocomposite film and an Al substrate; *E* and *h* denote the elastic modulus and thickness, respectively; subscripts *s* and *f* represent the substrate and film, respectively.

The displacement $\left(\delta_{DC}\right)$ at *D* can be divided into two components. One of the displacements ($\delta_{DC,1})$ is induced by the bending of the beam *DC*, and the other displacement $\left(\delta_{DC,2}\right)$ is caused by the rotation at point *C*.

(5) $$\delta_{DC}=\delta_{DC,1}+\delta_{DC,2}\;\;$$

(6) $$\delta_{DC,1}=\frac{Fa^{3}}{6\left(E_{f}I_{f}+E_{s}I_{s}\right)}$$

(7) $$\delta_{DC,2}=\frac{Fa}{4\bar{E}\bar{I}}\left(L^{2}-a^{2}\right)$$

The displacement at point *B* where the load is exerted can be calculated by substituting Equations (2), (3) and (5)–(7) into Equation (1).

(8) $$\Delta =\frac{Fa^{3}}{12\left(E_{f}I_{f}+E_{s}I_{s}\right)}+\frac{F\left(2L^{3}-a^{3}\right)}{12\bar{E}\bar{I}}$$

The work exerted on the ENF specimen by the external load *F* is ready to be calculated as follows.

(9) $$W=\frac{1}{2}F\;\Delta =\frac{F^{2}}{24}\left(\frac{a^{3}}{E_{f}I_{f}+E_{s}I_{s}}+\frac{2L^{3}-a^{3}}{\bar{E}\bar{I}}\right)$$

Thus, the mode II strain energy release rate can be obtained as follows.

(10) $$G_{II}=\underset{\delta A\to 0}{\lim}\left|\frac{\delta W}{\delta A}\right|=\underset{\delta a\to 0}{\lim}\left|\frac{\delta W}{b\delta a}\right|=\frac{F^{2}a^{2}}{8b}\left(\frac{1}{E_{f}I_{f}+E_{s}I_{s}}-\frac{1}{\bar{E}\bar{I}}\right)$$

**Figure 4:**
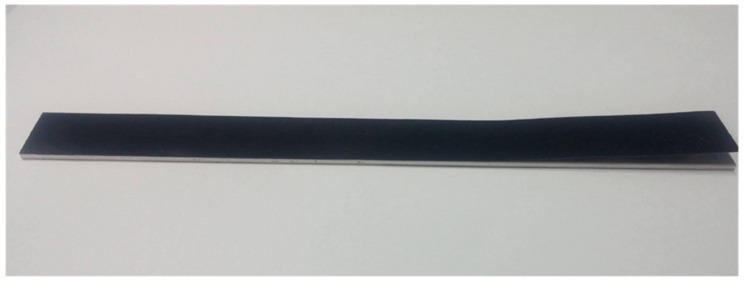
ENF specimen with an interfacial edge crack.

**Figure 5:**
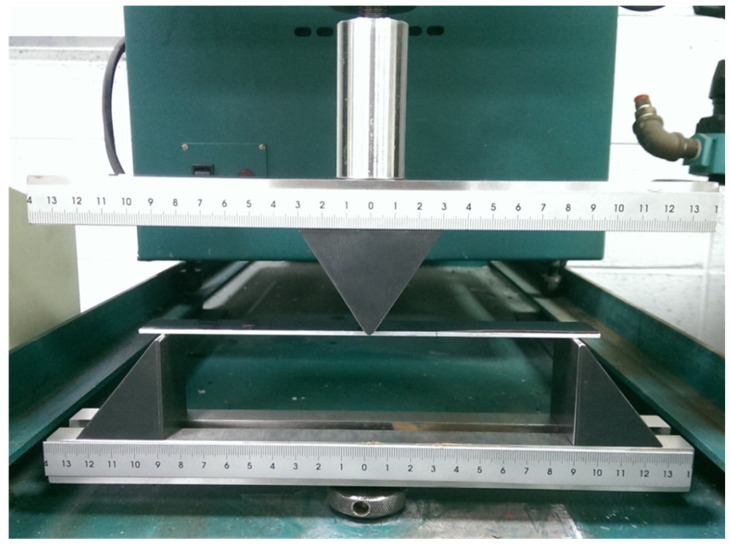
Experimental setup of three-point bending test on an ENF specimen.

In this work, the dimensions of the ENF specimen were length 180 mm and width 19 mm. The thicknesses of the nanocomposite film and substrate were 0.3 mm and 2 mm, respectively. The interfacial edge crack length was 60 mm. The as-prepared ENF test specimen is shown in Figure 4. The ENF specimen was under a three-point bending loading, as shown in Figure 5. The load was slowly increasing at a loading rate of 0.5 mm/min to reach a critical load$\;F_{cr\;}$, which initiates the crack propagation. Substituting the critical load$\;F_{cr}$ into Equation (10) leads to the determination of critical mode II strain energy release rate $G_{IIc}$. Three ENF test specimens were fabricated and performed the three-point bending tests to determine the mode II fracture toughness for each GNP content. The average of the three test results was presented.

**Figure 6:**
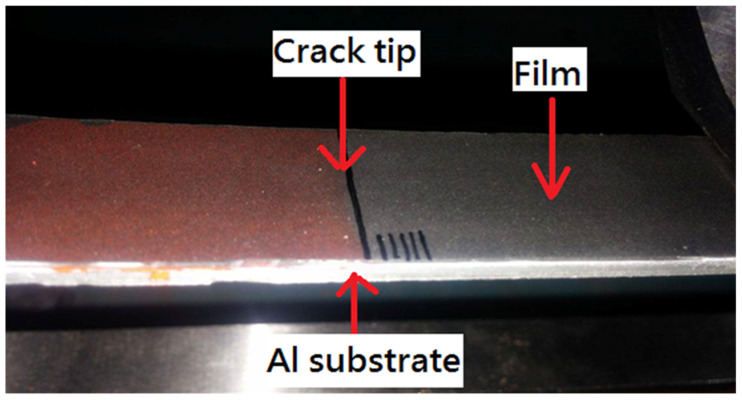
Red ink infiltrated into interfacial crack region.

**Figure 7:**
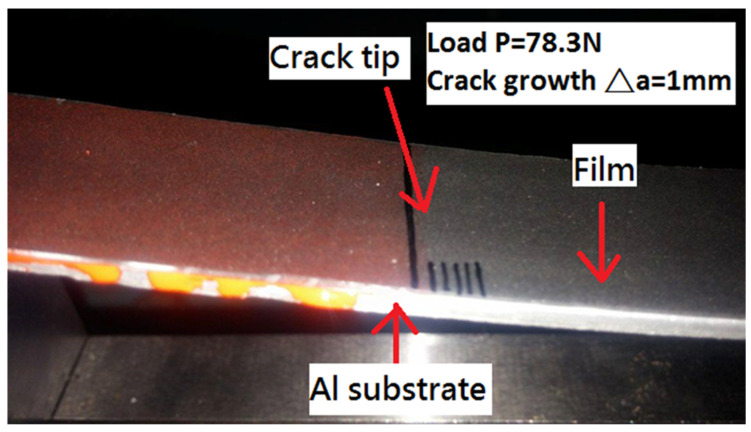
Initiation of crack growth at critical load.

Since GNP-reinforced nanocomposite appears in a black color even at a very small amount of GNP, it is difficult to identify the crack tip. To resolve this problem, red ink was infiltrated into the interfacial crack region, as shown in Figure 6. The contrast in colors makes the detection of crack propagation easier. Figure 7 illustrates the initiation of the crack propagation at the critical load $F_{cr}$.

## 3. Results and Discussion

### 3.1. Surface Morphology

**Figure 8:**
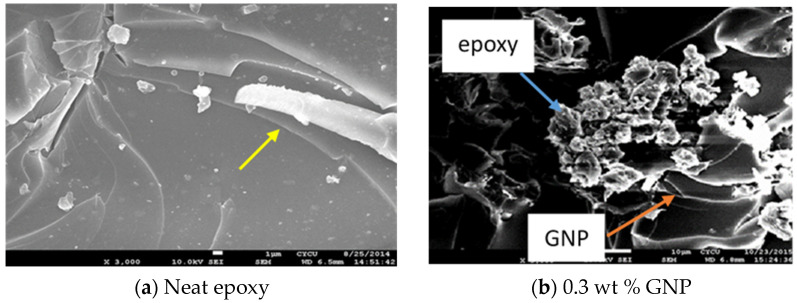

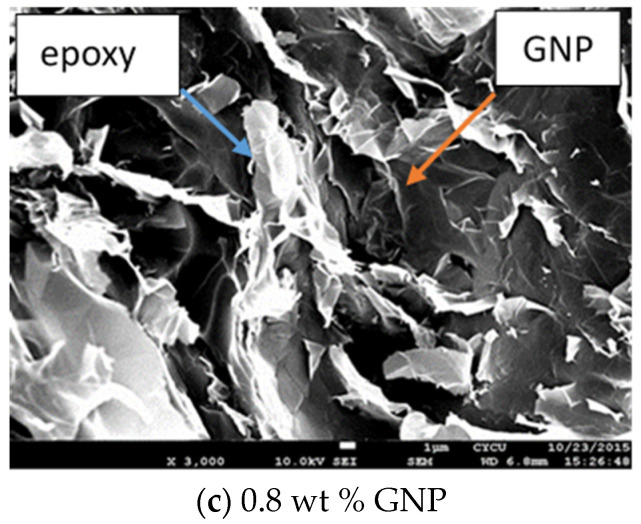
SEM images of surface morphologies (**a**) neat epoxy, (**b**) nanocomposite reinforced with 0.3 wt % GNP, (**c**) nanocomposite reinforced with 0.8 wt % GNP.

Carbon-based nanomaterials including CNT and GNP exhibit a tendency of agglomeration due to van der Waals forces and π−π bonding. GNPs dispersion in the epoxy matrix has a significant influence on the performance of the GNP-reinforced nanocomposite. SEM images of the surface morphologies for the neat epoxy and nanocomposites incorporated with 0.3 wt % and 0.8 wt % of GNPs are presented in Figure 8a–c, respectively. It appears that the neat epoxy exhibits a smooth surface morphology in conjunction with “river-like” fracture patterns indicated by a white arrow in Figure 8a. The area between the “river-like” patterns is very smooth, depicting a rapid crack growth. This demonstrates a typical brittle characteristic of the neat epoxy. Qiu and Wang [35] also reported a “river-like” fracture surface for the neat epoxy. In contrast, a coarser surface was observed for the nanocomposites reinforced with GNP, as shown in Figure 8b,c. GNP exhibits a wrinkle-like texture, resulting in an increase of the surface roughness [44]. GNPs were decorated with stacks of epoxy pointed by a green arrow in Figure 8b,c. The debonding did not occur at the interface between the GNP and epoxy matrix. This illustrates that there is a strong interaction between the GNP and epoxy. Moreover, SEM images illustrate that GNPs were well dispersed in the epoxy resin.

### 3.2. Hardness and Young’s Modulus

**Figure 9:**
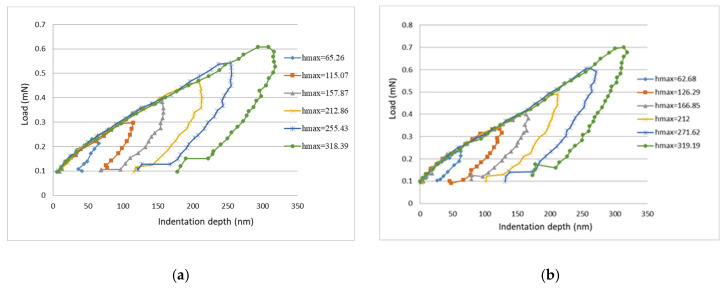

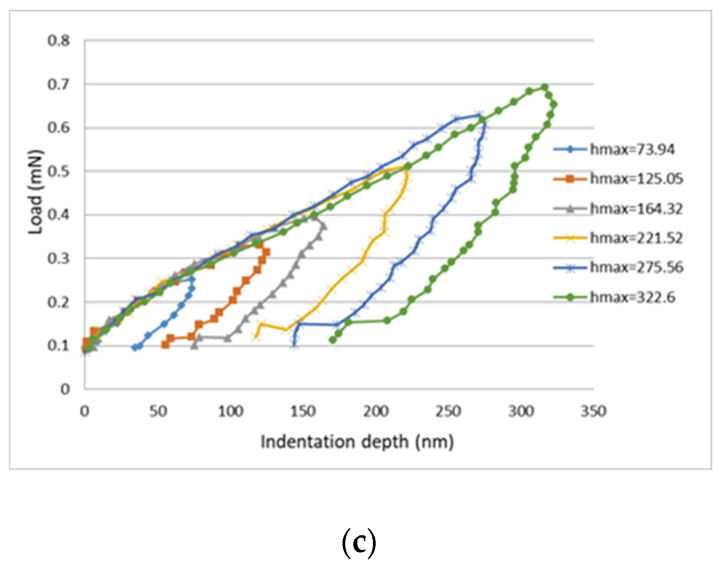
Load vs. indentation depth curves for nanocomposite films. (**a**) neat epoxy (**b**) nanocomposite reinforced with 0.3 wt % GNP, (**c**) nanocomposite reinforced with 0.8 wt % GNP.

**Figure 10:**
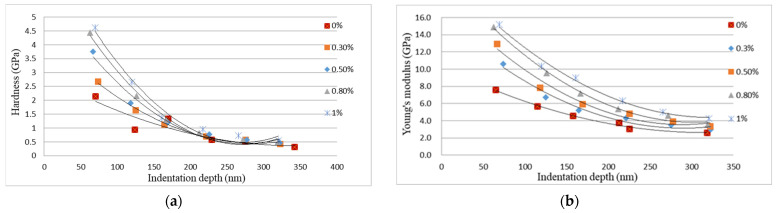
Hardness and Young’s modulus of the nanocomposite films reinforced with different loadings of GNP. (**a**) Hardness vs. indentation depth (**b**) Young’s modulus vs. indentation depth.

The load vs. indentation depth curves from the nanoindentation tests for neat epoxy and nanocomposites reinforced with 0.3 wt % and 0.8 wt % of GNPs are plotted in Figure 9. There are six curves for each specimen, which correspond to six different indentation depths ranging from 60 to 320 nm. Utilizing the continuous stiffness mode provided by the nanoindentation test instrument, the hardness and Young’s modulus of the nanocomposite film can be measured as a function of indentation depth. Figure 10 shows the hardness and Young’s modulus of the nanocomposites with different GNP concentrations varying with indentation depth. Table 1 presents the hardness and Young’s modulus of nanocomposite films incorporated with various contents of GNPs evaluated at the indentation depth of 320 nm. It can be observed that the hardness and Young’s modulus are improved by 71.8% and 63.2%, respectively, in comparison with neat epoxy.

The reason for the enhancement of the hardness and Young’s modulus can be attributed to local constrains, which were generated due to the presence of much stiffer nanofillers GNPs in comparison with the epoxy matrix. GNPs act as hard nanofillers and are uniformly distributed in and react with the epoxy matrix, thereby strengthening the GNP-reinforced nanocomposite. In contrast, poor dispersion causes the formation of micro voids and GNPs agglomeration, leading to stress concentration and slippage of the overlapped GNPs. As a result, the load transfer from the GNP to the epoxy matrix is reduced, and there is a degradation of mechanical properties of the nanocomposite. Thus, the improvements of the hardness and Young’s modulus of the nanocomposite film can be attributed to the strengthening effect by GNP in conjunction with strong interfacial interaction between GNPs and the epoxy matrix, as shown in Figure 8b,c.

Moreover, the hardness and Young’s modulus of the nanocomposite are decreasing with the increase of the indentation depth. In the indentation tests, the indentation depth is increasing with the increase of the indentation load. As the indentation depth increases, the plastic deformation of the nanocomposite film is increasing. Thus, the hardness and Young’s modulus of the nanocomposite film are decreasing as the indentation depth is increasing due to the increase of the plastic deformation.

### 3.3. Mode II Strain Energy Release Rate

**Figure 11:**
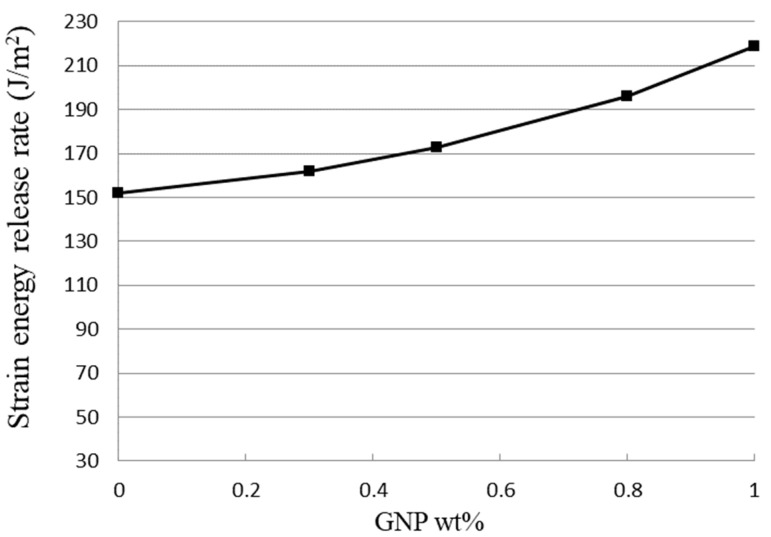
Mode II fracture toughness of nanocomposite film/substrate composite structure reinforced with various GNP contents.

Analytical expression of the mode II interfacial fracture toughness of the film/substrate composite structure with an interfacial edge crack has been derived in Section 2.4. Three-point bending tests were performed on ENF specimen to experimentally determine the mode II strain energy release rate. The experimental results of the mode II fracture toughness for the film/substrate composite structures with various GNPs contents are presented in Table 2. It can be seen that the mode II strain energy release rate of the nanocomposite film on the Al substrate composite structure is increasing with the increase of GNP loading, as shown in Figure 11. This demonstrates the effectiveness of GNP in improving the mode II interfacial fracture toughness of the nanocomposite. The strain energy release rate of the nanocomposite reinforced at a GNP content of 1.0 wt % exhibits an increase of 44.4% in comparison with pure epoxy.

The strain energy release rate is considered as a measurement of the energy required to induce an extension of the crack, which is crucial to evaluate the structural safety. A two-dimensional geometry of GNP has a very large aspect ratio. This feature provides an important mechanism for the fracture resistance referred to as crack bridging [45]. A bridging process of a matrix crack makes the nanocomposite more resistant to the crack propagation. In addition, the waviness and curved shape of GNP hinder the crack propagation and prompt crack deflection, resulting in a higher fracture resistance [46]. Two other important factors contributed to the toughening effect of GNP are (1) good dispersion of GNP in the epoxy matrix and (2) strong interfacial adhesion between the GNP and epoxy. The SEM images shown in Figure 8b,c depict a good dispersion and interaction of GNP in epoxy resin. Wang et al. [32] also reported that GNP can effectively disturb and deflect the crack propagation due to its two-dimensional structure in combination with a large aspect ratio.

A smooth surface morphology of the neat epoxy as shown in Figure 8a illustrates a brittle fracture behavior with weak resistance to the crack propagation [47]. On the other hand, nanocomposite incorporated with GNPs exhibits a rough surface. It is noted that an increase of the surface roughness is induced by the creation of plastic deformation [48]. As a result, a large fracture energy is dissipated. Furthermore, GNP with a large surface area and strong interfacial adhesion with epoxy matrix can bridge microcracks and prevent the crack propagation. It can be concluded that good dispersion in conjunction with strong adhesion between GNPs and the epoxy matrix leads to an increase of energy dissipation and effectively alters the crack propagation during the fracture process.

## 4. Conclusions

In this study, nanocomposite films containing 0.3, 0.5, 0.8 and 1.0 wt % of GNPs were fabricated and deposited on an Al substrate through a spinning coating process. The hardness and Young’s modulus of the nanocomposite film were determined by a nanoindentation test. In addition, the mode II interfacial fracture toughness of the nanocomposite film on Al substrate was evaluated using an ENF test. Experimental results show that the hardness, Young’s modulus, and mode II fracture toughness of the nanocomposite film are increasing with the increase of GNP content. The improvements of the hardness, elastic modulus, and mode II fracture toughness of the nanocomposite film incorporated with 1 wt % of GNP were 71.8%, 63.2% and 44.4%, respectively, in comparison with neat epoxy. SEM images demonstrate good dispersion and strong interaction between the GNPs and epoxy matrix, leading to the enhancements of the mechanical properties of the nanocomposite film.

## Figures and Tables

**Table 1:** Hardness and Young’s modulus of nanocomposite films incorporated with various contents of GNPs evaluated at the indentation depth of 320 nm.

GNP Contents	Hardness (GPa)	Young’s Modulus (GPa)
0 wt %	0.32	2.61
0.3 wt %	0.42	2.98
0.5 wt %	0.46	3.36
0.8 wt %	0.49	3.55
1.9 wt %	0.55	4.26

**Table 2:** Mode II fracture toughness of the nanocomposite film/substrate composite structure with various GNP contents.

GNP wt %	Specimen 1		Specimen 2		Specimen 3		Average Strain Energy Release Rate $G_{II}$ (J/m^2^)
	Critical Load $F_{cr}$ (N)	Strain Energy Release Rate $G_{II}$ (J/m^2^)	Critical Load $F_{cr}$ (N)	Strain Energy Release Rate $G_{II}$ (J/m^2^)	Critical Load $F_{cr}$ (N)	Strain Energy Release Rate $G_{II}$ (J/m^2^)	
0%	70.2	152.34	69.9	151.22	70	152.14	151.9 ± 0.68
0.3%	78.3	162.69	78.1	162.15	78.2	162.45	162.43 ± 0.28
0.5%	83.3	173.41	83.3	173.41	83.2	173.28	173.37 ± 0.08
0.8%	86.5	196.50	86.5	196.50	86.2	195.85	196.28 ± 0.43
1%	89	219.68	89	219.68	88.5	218.88	219.39 ± 0.51

## Data Availability

Data available on request.

## Nomenclature

Δ	displacement at the middle point of ENF test specimen
$\delta_{AB}$	displacement at left end of ENF test specimen
$\delta_{CB}$	displacement at crack tip of ENF test specimen
$\delta_{DC}$	displacement at right end of ENF test specimen
$\bar{E}\bar{I}$	flexural rigidity of the composite beam
$E_{f}$	Young’s modulus of the film
$E_{s}$	Young’s modulus of the substrate
$h_{f}$	thickness of the film
$h_{s}$	thickness of the substrate
a	crack length
$G_{II}$	mode II strain energy release rate
$F_{cr}$	critical load
$G_{IIc}$	critical mode II strain energy release rate
